# Medical Simulation-Based Learning Outcomes in Pre-Clinical Medical Education

**DOI:** 10.7759/cureus.11875

**Published:** 2020-12-03

**Authors:** Maxwell J Jabaay, Dario A Marotta, Stephen L Aita, Dianne B Walker, Leah O Grcevich, Victor Camba, James R Nolin, James Lyons, John Giannini

**Affiliations:** 1 Department of Research, Alabama College of Osteopathic Medicine, Dothan, USA; 2 Department of Neurology, Division of Neuropsychology, University of Alabama, Birmingham, USA; 3 Department of Psychiatry, Geisel School of Medicine at Dartmouth, Lebanon, USA; 4 Department of Simulation, Alabama College of Osteopathic Medicine, Dothan, USA; 5 Department of Clinical Sciences, Alabama College of Osteopathic Medicine, Dothan, USA

**Keywords:** undergraduate, pre-clinical, medical education, comlex, usmle, emergency medicine, clinical

## Abstract

Introduction

Medical simulation is widely used in the United States medical curriculum. However, learning outcomes based on simulation have yet to be reported. In this study, we aim to characterize the objective performance of first- and second-year medical students following eight weeks of medical simulation-based learning.

Methods

First- (n=25) and second-year (n=15) medical students were recruited for this study. We designed and administered a novel pre-experience examination to collect participant demography and assess simulation and non-simulation knowledge. Following 14 high-fidelity simulation scenarios over the course of eight weeks, we administered an identical post-experience examination and compared performance, primarily using a within-subjects analytic design.

Results

Student performance improved by an average of 18% following the medical simulation experience, and first-year students demonstrated greater benefit (22%) as compared to second-years (12%). Relative to first-years, second-year students showed higher overall performance on both pre- and post-examination. Demographic factors and prior medical experience were not significantly associated with assessment performance and score improvement.

Conclusions

Our data supported the efficacy of simulation-based learning as evidenced by the significant improvement in objective performance on a standardized examination. That is, both first- and second-year medical students demonstrated test-score improvement following an eight-week medical simulation program. Of note, the first-year students exhibited greater benefit (at the group level). Importantly, these findings were statistically unrelated to participant demographic and background variables. Collectively, this study provides preliminary evidence that medical simulation in the pre-clinical phase of undergraduate medical education is an effective tool for student learning.

## Introduction

Medical simulation-based learning is a multidisciplinary approach to medical learning by which subject matter experts produce realistic medical scenarios to facilitate learning in a controlled setting [1]. Contemporary simulation encourages active learning, reinforces didactic material, and presents clinically relevant topics as an authentic assessment of medical knowledge [2-4]. Simulation learning also provides an engaging environment in which students can practice high-stress medical decision making without jeopardizing patient safety [5-6]. As such, simulation training provides a pragmatic and affordable way to implement standardized patient-care experiences earlier in a student’s medical education [7].

Traditionally, pre-clinical US medical education has been carefully tailored to prepare students for the first round of medical licensing examinations, which are highly correlated to residency placement and largely focus on the fundamentals of biomedical sciences [8-9]. In an unprecedented advance, the United States Medical Licensing Examination (USMLE) Step 1 will transition from a nominal score value to a dichotomous “pass-fail” scoring system beginning in the year 2022. In response to this change, residency program directors report that an increased weight will be placed on the score of USMLE Step 2 exams, which emphasize clinical management [10]. Commensurate with this change, we expect to see an earlier introduction of clinical experiences to the medical school curriculum. However, this is problematic given the competition between interprofessional students requiring preceptorships and advances in medical management have led to decreasing lengths of stay in the hospital. As such, there are fewer opportunities for gaining clinical experience than ever before [11]. Therefore, the exploration of simulation modeling into pre-clinical education may provide a viable solution to introducing clinical experience amidst a shortage of qualified clinical preceptorships.

There is an extensive body of literature discussing the effectiveness of low- and high-fidelity simulation in clinical practice to enhance the ecological validity of medical instruction [12-15]. Despite this, there is a paucity of studies directly examining the utility of simulation learning in the pre-clinical phase of medical education. More specifically, the current literature lacks a detailed comparison of simulation learning outcomes between first- and second-year medical students. To address this gap in the literature, we investigated the effects of an eight-week medical simulation experience between first- and second-year medical students. We also chose to evaluate the impact of a student’s background (ie., demographics, year in medical school, and past medical experiences) on simulation learning. We predicted that first- and second-year medical students would benefit, albeit differently, from participation in medical simulation, as assessed by score improvement on a standardized examination. We also hypothesized a relation between past student experiences (medical, educational, and simulation) and examination performance such that these modifying factors would produce greater improvement in post-test scores compared to students without past medical experiences or fewer years in medical school.

## Materials and methods

Participants

A total of 40 medical students at a college of osteopathic medicine located in the southeastern United States, voluntarily participated in an eight-week high-fidelity medical simulation program between September 2019 and November 2019. Participants were limited to first- and second-year medical students. This study was approved by the college’s institutional review board (IRB# HS190820EX). Written consent was obtained separately from each participant prior to the administration of pre- and post-simulation examinations.

Data collection

Assessment

Within one week from the beginning of the simulation learning experience, participants were administered a 31-question multiple-choice pre-test designed to assess the following: 1) six questions assessing demographic variables; 2) eight medically related questions that were not reviewed during the simulation experience (note: these questions served a ‘control’ function where they were purposely unrelated to the simulation experience to account for/measure artifact score change); 3) 16 medically-related questions whose content was taught during the simulation experience, which served as a measure of simulation performance. Test questions were created by study authors based on the specific cases used during the eight-week simulations. Participants were not given feedback or answers to test questions. Scores were de-identified using a two-digit number, which was only known by the participant. This number was used to match pre- and post-test scores for comparison.

Participants were not informed of the existence of the post-test until just before the post-test was administered. A post-test, composed of identical questions as the pre-test, was administered less than one hour after completing the final training case. One medically related, non-simulation question was inadvertently released to participants during a debriefing session (prior to the post-test); consequently, we excluded this item from all analyses. See Appendix A for copies of the pre- and post-simulation examinations.

Simulation Experience

Participants were self-arranged into eight groups of five participants, each of who met weekly for the simulation experience. Each participant was assigned a specific role for each simulation patient encounter (e.g., team captain, scribe, history taker, physical examiner, and case coordinator). Students were allowed to change roles within groups during subsequent encounters.

In each weekly simulation experience, two groups met at the host institution’s simulation complex. Participants were not provided any information before each encounter. One group actively participated in an acute care scenario featuring a high-fidelity human patient simulator programmed using simulation software. The remaining group observed the unfolding scenario via live remote audio and video streaming to the debriefing room. The observation group was provided with the same laboratory values and imaging by a second clinical simulation instructor to keep the observation group engaged during the patient encounter.

During the case, participants were presented with the patient’s chief concern, medications, past medical history, family history, and social history to varying degrees of detail. Participants were not given explicit instructions on how to work as a team to obtain relevant history and physical findings. Instead, they were given full autonomy. Participants could obtain the history from the patient or caregiver, perform any physical examinations, order laboratory tests and imaging, perform procedures, administer medication with real-time feedback, and order results pushed to an in-room monitor. Scenarios were programmed to progress through a variety of stages based on ideal management strategies. Each scenario lasted for approximately 15 minutes and was concluded at the discretion of the instructor. Immediately following the scenario, clinical simulation instructors conducted a debriefing session with both participant groups. The debriefing session consisted of an interactive 20-minute discussion summarizing key aspects of the case such as patient presentation, laboratory and imaging findings, and ideal patient management strategies. At the conclusion of the debriefing session, the two participant groups switched roles and experienced a second unrelated encounter. Each team participated in a supplementary voluntary session, which reviewed the capabilities of the high-fidelity simulator, including heart sounds, lung sounds, dysrhythmias, and supplies available in the crash cart; this was an adjunctive experience in which no team practiced an additional vignette.

In total, each group experienced seven encounters via direct participation and seven encounters via observation for a total of 14 experienced simulation cases. A summary of the 14 clinical vignettes is available in Appendix B.

Measures

The demographic variables - sex, class year (e.g., 1st year, 2nd year), the highest educational level achieved, prior medical experience, and prior competitive simulation experience - were collected as part of the pre- and post-test examination.

Performance variables: Pre- and post-test scores were used as performance metrics. Data were compiled and entered into Qualtrics computer software, which collated and parsed the data into desired outputs. An independent manual tabulation of data was also conducted to confirm the data was collected and recorded correctly.

SIM score: Sixteen medically related multiple-choice questions associated with simulation cases served as a measure of cohort simulation performance. SIM questions are generally related to the following content areas: basic knowledge, cardiology, case management, neurology pathology, pharmacology, respiratory, and trauma. 

Non-SIM score: Eight medically related multiple-choice questions not related to cases covered during the simulation served as a control.

Change score: Differences in the post- and pre-test SIM and non-SIM scores were tabulated to assess a participant’s relative change in performance. Separate change variables for the SIM and non-SIM scales were computed as follows: Change (Δ) Score = Post-Test Score - Pre-Test Score. Accordingly, positive values on change variables reflect score increase while negative values indicate an interval score decrease.

Statistical analysis

Prior to conducting main analyses, demographic and performance variables were examined for group differences (i.e., between first-year and second-year students) using chi-squared (χ2) tests for categorical variables and independent t-tests for continuous variables. In addition, performance variable distributions were examined for normality (i.e., skewness and kurtosis). Lastly, bivariate correlations were performed to determine whether demographic variables were associated with any of the performance-based outcome variables for the total sample.

For main analyses, paired-samples t-tests were performed on SIM and non-SIM-related questions to evaluate pre-post score differences following the eight-week simulation experience. The analysis was conducted across the total sample as well as within the first- and second-year students (separately) to examine the consistency of the effects. Finally, the degree of test-score change was alternatively assessed using a computed variable of score change for each performance-based variable. Doing so allowed for direct comparison of first- and second-year medical students’ score change for SIM and non-SIM-related evaluative questions.

The main measure of effect size for t-tests was Cohen’s d, with values of 0.2, 0.5, and 0.8 corresponding to small, medium, and large effect sizes, respectively [16]. Additional effect size indices interpreted were Cramér’s V (φc) for χ2 tests and Pearson’s r correlation coefficient for bivariate correlations; values were interpreted similarly, where 0.1, 0.3, and 0.5 reflected weak, moderate, and strong associations, respectively [16]. The level of statistical significance (α) was set to 0.05 (two-tailed) for all analyses. Participants with missing or improperly completed examinations were excluded from the analyses. The Statistical Package for the Social Sciences (SPSS) program, version 26 (IBM Corp., Armonk, NY) was used for all statistical analyses.

## Results

Sample characteristics

Table 1 displays demographic and performance variable descriptive statistics for the final total sample as well as stratified by medical student year. From the original sample of 40 participants, three (7.5%) were excluded from data analyses due to missing post-test scores (n = 2, 5%) and having to repeat first-year coursework (n=1, 2.5%). The majority of final samples had attained a bachelor’s degree as the highest level of education (n=28, 75.7%), were male (n=25, 67.6%), and were first-year medical students (n=22, 59.5%) who possessed some level of prior medical experience (n=28, 75.7%). Overall, prior medical experience consisted mostly of being a prior medical scribe (n=16; 43%), followed by nursing assistant (n=8; 22%), emergency medical technician (n=4; 11%), nurse or nurse practitioner (n=2; 5%), and pharmacist (n=1; 3%). Demographic comparisons between first-year (n=22) and second-year (n=15) students revealed no significant difference in gender [63.6% vs. 73.3% male; χ2(1, n=37)=0.38, p=.54, φc=.10], educational attainment [72.7% vs. 80.0% bachelor’s; χ2(2, n=37)=0.78, p=.68, φc=.15], and prior history of medical experience [77.3% vs. 73.3%; χ2(1, n=37)=0.08, p=78, φc=.05].

**Table 1:** Descriptive statistics for demographic and performance variables Note. es=effect size; p values for demographic variables reflect chi-square analyses, and independent t-tests for the test variables; respective effect size statistics shown in parentheses for each set of variables. ^a^ SIM refers to simulation-related examination questions exclusively. ^b^ Non-SIM refers to non-simulation-related examination questions exclusively. ^c^ SIM and non-SIM score change, denoted by “Δ,” was computed as [Post-Test Score – Pre-Test Score], where positive values reflect interval increase in test-scores.

Variable Type	All Participants (n = 37)	1^st^ Year Medical Students (n = 22)	2^nd^ Year Medical Students (n = 15)	p (es)*
Demographic Variables				(φ_c_)
Sex, n(%)				
Female	12(32.4)	8(36.4)	4(26.7)	.38 (.10)
Male	25(67.6)	14(63.6)	11(73.3)	
Education Level, n(%)				
Bachelor’s Degree	28(75.7)	16(72.7)	12(80.0)	.68 (.15)
Master’s Degree	8(21.6)	5(22.7)	3(20.0)	
Doctorate	1(2.7)	1(4.5)	0(0.0)	
Past Medical Experience, n(%)				
Yes	28(75.7)	17(77.3)	11(73.3)	.78 (.05)
No	9(24.3)	5(22.7)	4(26.7)	
Medical Experience Type, n(%)				
Medical Scribe	16(43.2)	10(45.5)	6(40.0)	N/A
Nursing Assistant	8(21.6)	5(22.7)	3(20.0)	
Emergency Medical Tech	4(10.8)	2(9.1)	2(13.3)	
Nurse	1(2.7)	0(0.0)	1(6.7)	
Nurse Practitioner	1(2.7)	0(0.0)	1(6.7)	
Pharmacist	1(2.7)	1(4.5)	0(0.0)	
Test Variables,M(SD) %Correct ^a,b^				(d)
SIM Pre-Test	9.6(2.6) 60%	8.4(2.1) 53%	11.4(2.4) 71%	< .001(1.33)
SIM Post-Test	12.5(2.2) 78%	12.0(2.6) 75%	13.3(1.4) 83%	.09(0.61)
SIM Score Δ^c^	2.9(2.3) 18%	3.6(2.3) 22%	1.9(1.9) 12%	.02(0.82)
Non-SIM Pre-Test	3.7(1.8) 46%	3.6(1.8) 45%	3.9(1.9) 48%	.66(0.15)
Non-SIM Post-Test	3.7(1.7) 46%	3.2(1.6) 40%	4.3(1.6) 54%	.05(0.69)
Non-SIM Score Δ^c^	0.0(1.4) 0%	-0.4(1.5) -5%	0.5(1.1) 6%	.07(0.64)

Performance variables

Visual inspection of performance variable distributions as well as skewness and kurtosis statistical tests were not concerning for non-normality. Accordingly, parametric statistical analyses were deemed appropriate. The total evaluation scale (i.e., all 24 items summed) demonstrated acceptable reliability, as measured by internal consistency, for both the pre- and post- administrations (Cronbach’s α= .72 and .76, respectively). Bivariate correlations were carried out to examine relations between demographic/background variables and performance outcome variables in the total sample. Education, gender, and prior medical experience were not significantly correlated with any of the SIM and non-SIM outcome variables. Next, independent t-tests were conducted to examine group differences in each performance variable. Analysis revealed first-year students produced significantly lower SIM pre-test scores [t(35)=-4.02, p<.001, d=1.33] and non-SIM post-test scores [t(35)=-2.05, p=.05, d=0.69)] compared to second-year students. See Table 1 for full descriptive statistical information.

SIM and non-SIM test score change

Table 1 displays SIM and non-SIM test score changes. Table 2 displays paired-sample t-test findings for pre- and post-test raw scores for the overall sample as well as by year in medical school. For the overall sample, significant pre-post test score change was observed for SIM [t(36)=-7.75, p<.001, d=1.27] but not non-SIM [t(36)=-0.12, p=.91, d=0.02] questions. We then conducted the same within-subjects analysis separately for each medical school year group. For first-year students, SIM test-scores significantly increased over the interval [t(21)=-7.38, p<.001, d=1.57], but there was no difference between pre- and post-scores on the non-SIM test [t(21)=1.16, p=.26, d=0.25]. Rather, the first-year students showed subtle non-SIM test-score decline following the interval (M declined from 3.6 to 3.2). Second-year medical students also showed improvement in SIM test-scores [t(14)=-3.84, p=.002, d=0.99] but not non-SIM [t(14)=-1.61, p=.13, d=0.41], following the eight-week simulation experience.

**Table 2:** Within-subjects t-test results for the total sample and stratified by medical student year ^a^ SIM refers to simulation-related examination questions exclusively. ^b^ Non-SIM refers to non-simulation-related examination questions exclusively.

Reference Group	Outcome Variable Pair^ a,b^	t	df	p	d
Total Sample	Pre- vs. Post-SIM Test-Score	-7.75	36	< .001	1.24
	Pre- vs. Post-Non-SIM Test-Score	0.12	36	.91	0.20
1st Year Medical Students	Pre- vs. Post-SIM Test-Score	-7.38	21	< .001	1.57
	Pre- vs. Post-Non-SIM Test-Score	1.16	21	.26	0.25
2nd Year Medical Students	Pre- vs. Post-SIM Test-Score	-3.84	14	.002	0.99
	Pre- vs. Post-Non-SIM Test-Score	-1.61	14	.13	0.41

Lastly, we investigated whether the degree of performance change differed as a function of the medical student group by way of computing a distinct score change variable for the SIM and non-SIM-related questions. Independent samples t-tests were then performed to compare SIM and non-SIM-related performance changes between first-year and second-year medical students. The analysis revealed a significant difference in score change for SIM-related questions [t(35)=2.42, p=.02, d=0.82] such that the first-year medical students demonstrated greater interval change in scores (M=3.6, SD=2.3) as compared to second-year students (M=1.9, SD=1.9). Alternatively, there was no significant group difference in non-SIM-related question score change [t(35)=-1.85, p=.07, d=0.64] though the second-year medical students displayed a mild trend of greater score improvement (M=0.5, SD=1.1) than the first-years (M=-0.4, SD=1.5).

## Discussion

Simulation learning in undergraduate medical education remains understudied, despite its potential role in providing low-risk clinical experiences to medical students. In this study, we used objective pre- and post-examination performance scores to compare first- and second-year medical students following an eight-week medical simulation experience. We found that both first- and second-year students had significant improvements in performance. While student scores improved overall, first-year students were found to have greater performance improvements compared to second-year students. Conversely, there were no significant differences attributed to a participant’s sex, education level, or past medical experience on performance. The lack of correlation found between the previously mentioned variables and percent change remained unmodified even after factoring in the student’s year in school. Medical students, irrespective of past experiences, demonstrated acquisition of knowledge over the interim period, likely reflecting a significant benefit from simulation learning experiences [17].

On average, medical students showed an improvement in performance of 18%. Performance improvement was twice as high in first-year students (22%) compared to second-year students (12%). This suggests high-fidelity medical simulation may be an effective learning tool in pre-clinical medical education, especially for first-year medical students. That is, the degree of benefit from the simulation experience may be moderated by medical student year, as first-year students benefited more than their second-year counterparts. Generally, students have a greater foundation of knowledge by the second year of medical school. For instance, at the host institution, first-year students had only experienced three months of basic science courses with minimal clinical instruction at the time of the study. Conversely, second-year students had completed system-based clinical courses in multiple body systems. As expected, second-year test performance was higher in both pre- and post- examinations. In a similar vein, a student’s year in medical school was positively associated with higher pre-test performance (r=0.56, p<0.01) such that second-year student status was strongly associated with higher pre-test scores. Meanwhile, a similar correlation was not observed with post-test performance. While first-year students scored 19% lower than second-year students on the pre-test, this deficit was attenuated to 8% on the post-test. Post-test scores for non-SIM items were not associated with the same degree of improvement but rather, there was a slight decline in scores following the eight-week training. Together, this improvement in first-year students may suggest medical simulation may help students assimilate a broader foundation of knowledge, especially in those who have less experience prior to medical school. 

In this study, 76% of participants had prior medical experience while 24% had a master’s degree or higher. Interestingly, neither of these attributes were associated with better post-test performance. This may be the result of context-dependent factors within the simulation scenarios themselves. For example, acute care settings, such as those simulated in these scenarios, require proficiency in situational awareness, medical decision-making, and overall clinical management to achieve desirable outcomes [18]. It is possible that medical experiences that are not directly related to acute care may not play a functional role in improving simulation-related performance. Similarly, even though a participant’s level of education may correlate to higher didactic performance, higher education levels may provide little value without being able to apply acquired knowledge within the confounds of a rapidly evolving medical scenario [19-20]. Since participant characteristics appear to not influence performance outcomes, students of varying backgrounds may find value in simulation learning. This is particularly important in the setting of undergraduate medical education, considering that the diversity of matriculating medical students in the United States has steadily risen over the past 40 years [21]. 

Several limitations are present in this study. First, this study only examined performance among first- and second-year medical students in the pre-clinical phase of medical education. Therefore, conclusions are not generalizable to third- and fourth-year students. Next, students were given identical pre- and post-examinations eight weeks apart, which introduces possible bias from practice effects. However, threats from this potential bias were remedied by the fact that (a) we analyzed interval change (lack thereof) in non-simulation based content (which all participants received at pre- and post-test periods), and (b) participants were not informed of the presence of a post-test to avoid recall bias in the form of purposeful memorization of questions and independently sought-out answers. Nevertheless, it remains a possibility that practice effects could have been differentially present in the question sets (e.g., stronger in simulation-related questions). Pertinent to our study, within-subject designs are also susceptible to subject history (i.e., participants having different life experiences across the study period) and maturation effects (i.e., participants differentially maturing over the interval period, which may influence performance. Order-effects and nonsymmetrical carry-over effects were not a concern by virtue of our study’s simulation intervention and identical tasks. Lastly, while an independent control group was not used as a part of the study design, an inherent strength of the within-subjects design is that individual differences are well-controlled (as participants act as their own controls).

## Conclusions

The results of this study demonstrate that preclinical medical students show significant improvement in objective performance measures following eight weeks of medical simulation. First-year students benefited most from the simulation experience, as evidenced by the highest change in post-test scores. Additionally, a student’s sex, the highest level of education, and prior medical experience had no bearing on performance outcomes. We predict that the changing of the USMLE Step 1 from a scored exam to pass-fail will shift medical education towards emphasizing early clinical contact. In this fluid environment, medical simulation serves to reinforce biomedical concepts and acts both as a teaching strategy as well as a tool to assess clinical competency. Together, this information supports the use of simulation learning in pre-clinical undergraduate medical education.

## Figures and Tables

**Figure 1:**
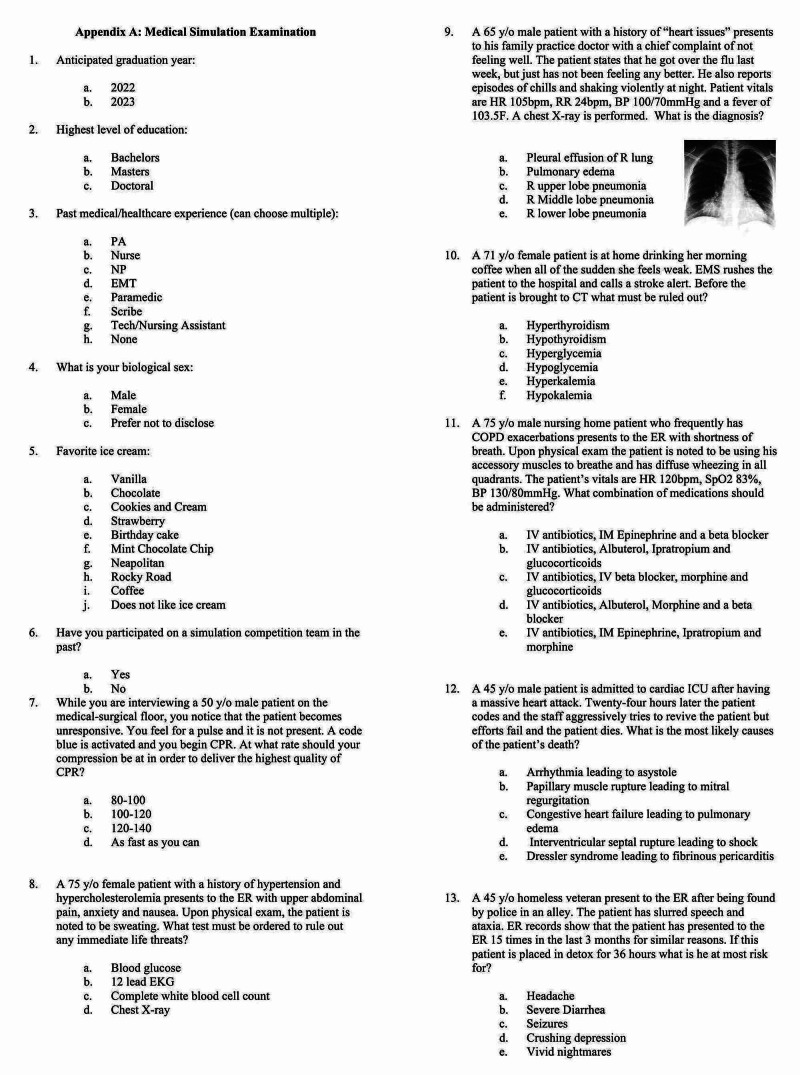
Written assessment part 1/3

**Figure 2:**
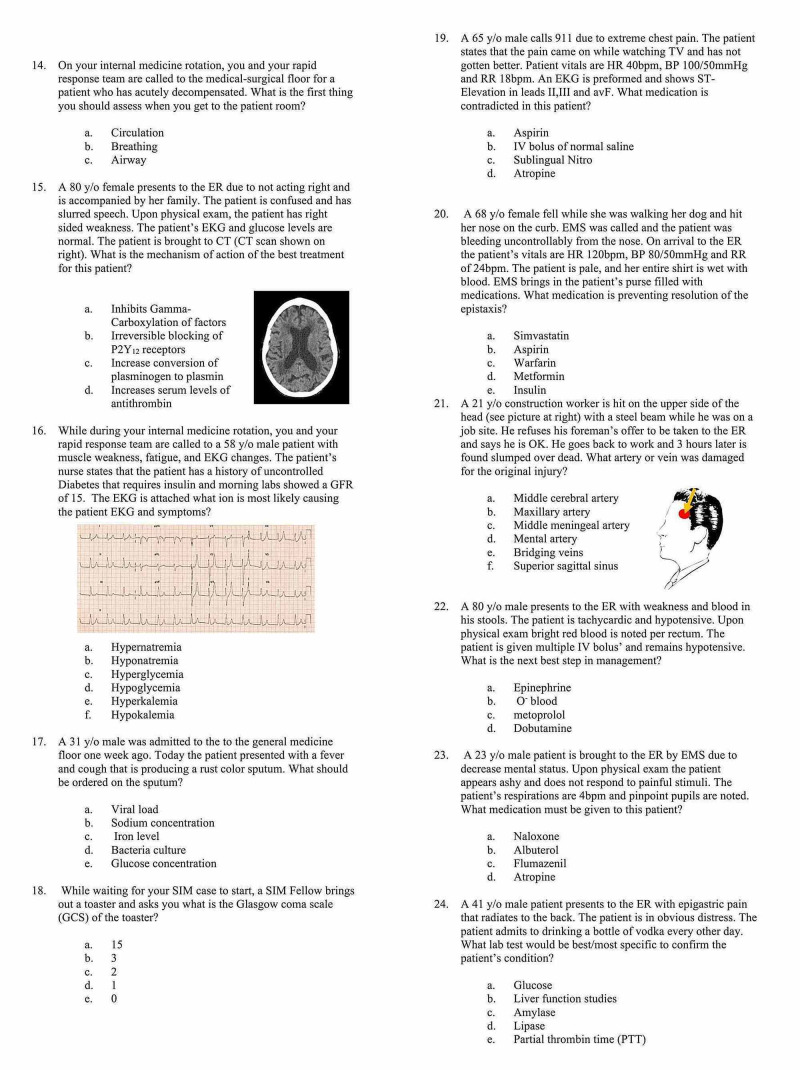
Written assessment part 2/3

**Figure 3:**
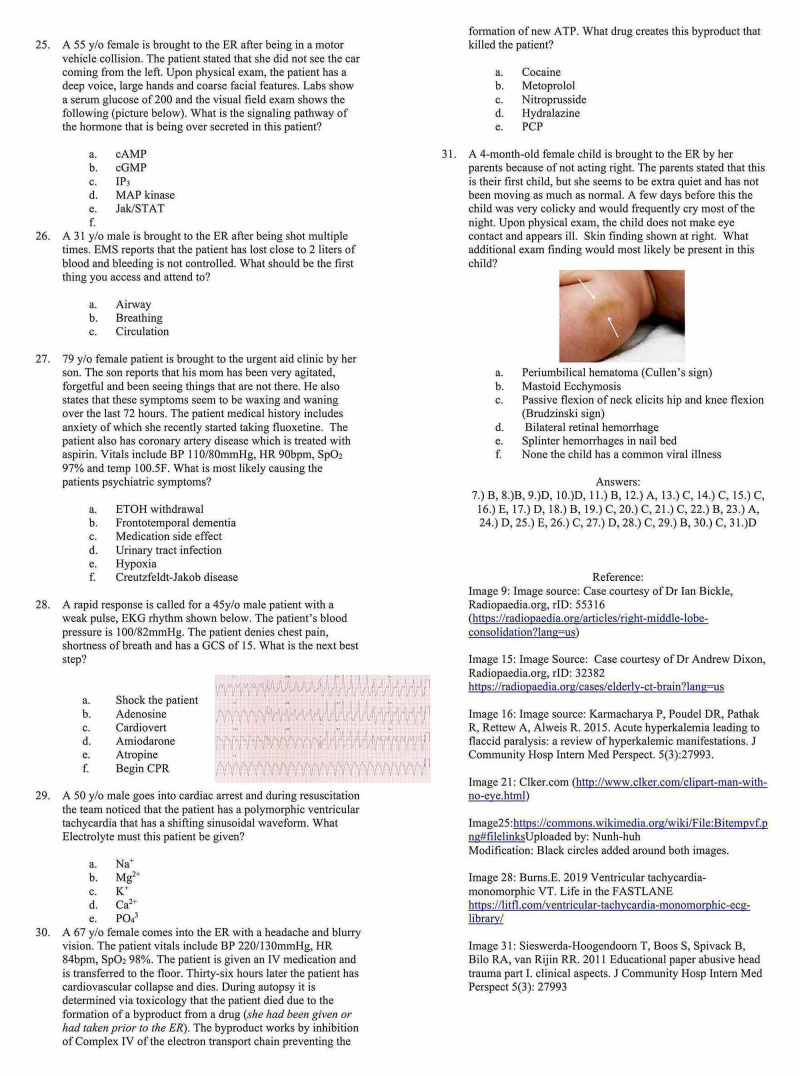
Written assessment part 3/3

## Appendices

Appendix A

Figure 1, Figure 2, and Figure 3 comprise the written assessment that was used as the pre-test and post-test.

Appendix B

**Table 3:** Simulation scenario summary

Simulation Scenario	Focus of Simulation Scenario
Orientation	Orientation
Scenario 1	Chronic obstructive pulmonary disease exacerbation (COPD)
Scenario 2	Atrial fibrillation
Scenario 3	Pulmonary embolism
Scenario 4	Ischemic stroke
Scenario 5	Neutropenia
Scenario 6	Hypertensive emergency
Scenario 7	Cardiogenic shock
Scenario 8	Serotonin syndrome
Scenario 9	Perforated ulcer
Scenario 10	Subdural hematoma
Scenario 11	Lupus complications
Scenario 12	Endocarditis
Scenario 13	Hypertensive emergency
Scenario 14	Hyperparathyroidism
